# Safety of Telemedicine Versus In-Person Care for Patients With Tracheal Devices: Propensity Score–Matched Cohort Study

**DOI:** 10.2196/95479

**Published:** 2026-05-26

**Authors:** Pedro Prosperi Desenzi Ciaralo, Paulo Francisco Guerreiro Cardoso, Helio Minamoto, Benoit Jacques Bibas, Carlos Roberto Ribeiro Carvalho, Paulo Manuel Pego-Fernandes

**Affiliations:** 1 Instituto do Coracao do Hospital das Clinicas da Faculdade de Medicina da Universidade de Sao Paulo, Rua Dr. Eneas de Carvalho Aguiar 44, São Paulo, 05403-904, Brazil, 55 35997035648

**Keywords:** telemedicine, digital health, electronic health records, tracheal diseases, airway stents, tracheostomy

## Abstract

**Background:**

Patients with tracheal diseases often require long-term follow-up after tracheal device placement, with a risk of adverse events that may lead to emergency care and unplanned interventions. Telemedicine has been proposed as an alternative to in-person follow-up to improve access and continuity of care.

**Objective:**

The primary objective of this study was to compare the need for emergency department (ED) visits between telemedicine and in-person groups. Secondary objectives included comparing hospital readmissions, 30-day hospital readmissions, and unplanned interventions between groups.

**Methods:**

This retrospective, single-institution study included adult patients with tracheal devices who underwent telemedicine and in-person outpatient clinic visits between 2020 and 2024. To balance the groups, we used 1:1 propensity score matching. We collected demographic and clinical data and evaluated the need for ED visits, hospital readmissions, 30-day hospital readmissions, and unplanned interventions. Kaplan-Meier estimation of time to first ED visit was performed to assess outcomes after outpatient visits.

**Results:**

A total of 483 patients (n=277, 57% telemedicine and n=206, 43% in-person) underwent 2487 visits (1258 telemedicine and 1229 in-person). After propensity score matching, 336 patients remained (168 in each group). There were no significant differences in the need for ED visits, hospital readmissions, or unplanned interventions. The telemedicine group had significantly fewer 30-day hospital readmissions (odds ratio 0.38, 95% CI 0.16-0.87; *P*=.02). Kaplan-Meier analysis indicated no statistically significant difference in ED-free visits.

**Conclusions:**

Telemedicine follow-up was associated with outcomes comparable to those of in-person follow-up in this cohort of adult patients with tracheal devices, with no evidence of an increased need for ED visits. In the matched analysis, telemedicine was associated with lower odds of 30-day hospital readmission.

## Introduction

Patients with tracheal devices frequently experience complications [1-3]. Clinical manifestations such as dyspnea, stridor, dysphonia, and infection are common [4], often leading to recurrent emergency department (ED) visits, hospital readmissions, and unplanned interventions [5].

These patients require continuous surveillance to ensure long-term safety and device functionality [6-9]. Proper stoma care, inspection of device integrity, and counseling on daily nebulizer use are essential components of follow-up [10,11]. Due to the chronic nature of tracheal diseases and the high risk of device-related complications, these patients demand prolonged and structured outpatient follow-up to maintain airway patency and stability.

In response to the limited number of specialized centers dedicated to the management of tracheal disease in Brazil [12] and anticipating an increase in the number of patients with tracheal disease following the COVID-19 pandemic [13-16] led us to develop a telemedicine program (TeleTrachea) [17,18] to maintain the support for this patient population. The implementation of TeleTrachea required establishing and standardizing new health care protocols and providing structured education for patients and physicians.

Despite the increasing use of telemedicine across medical specialties [19,20], no studies have addressed the safety of telemedicine follow-up in patients with tracheal devices. This is an important knowledge gap in this vulnerable patient population that requires continuous surveillance to prevent potentially life-threatening complications.

The lack of evidence regarding the safety and effectiveness of telemedicine in this context motivated this study. The primary outcome was the need for ED visits, and the secondary outcomes included hospital readmissions, 30-day hospital readmissions, and unplanned interventions.

## Methods

### Study Protocol

This is a retrospective cohort study of adult patients wearing tracheal devices who were followed at the Trachea and Airway Outpatient Clinic of the Division of Thoracic Surgery, Hospital das Clinicas, University of Sao Paulo between August 2020 and August 2024. All patients were identified through routine in-person follow-up at the tracheal disease team’s outpatient clinic. At the end of each face-to-face consultation, telemedicine follow-up was systematically offered by the attending physician. Patients who declined telemedicine were scheduled for continued in-person follow-up. For those who accepted telemedicine, the patient’s telephone number and institutional registration number were forwarded to the support team of the telemedicine department at the Heart Institute of Hospital das Clinicas, University of Sao Paulo (InCor-HCFMUSP).

The support team contacted the patient by telephone to arrange the telemedicine appointment, including the consultation date and time. Before the visit, the team provided instructions on using the platform and conducted audio and video tests to reduce the likelihood of technical issues during the teleconsultation. After the telemedicine visit, the patient was again referred to the support team to coordinate follow-up care, including scheduling return visits, imaging studies, prescriptions, medical reports, and endoscopic procedures, as indicated.

Patients were categorized into the telemedicine or in-person group, and follow-up modality remained unchanged throughout the study period. Patients in the telemedicine group were followed exclusively through telemedicine, whereas those in the in-person group were followed exclusively through face-to-face visits. In the telemedicine group, in-person hospital attendance occurred only when necessary for elective surgery or endoscopic procedures, or complementary diagnostic testing, excluding visits related to study outcomes.

Telemedicine follow-up was systematically offered to all patients evaluated during the in-person visit. Ongoing in-person appointments were maintained for patients who reported limitations to telemedicine, such as a lack of internet access, the absence of a caregiver to assist during the virtual visit, or a clear preference for in-person follow-up. A telemedicine consultation was considered complete when there was uninterrupted synchronous audio and video interaction between the physician and patient.

### Ethical Considerations

The study was approved by the research ethics committee for human subjects of InCor-HCFMUSP (protocol number 553422113). Given the retrospective design of the study and the use of data from electronic medical records, the ethics committee waived the requirement for informed consent.

Data were collected from the institution’s electronic health records. Access to identifiable information was restricted to the principal investigator (PPDC), who extracted the data and provided the study team with a fully anonymized database. Deidentification was performed using each patient’s institutional enrollment number in accordance with institutional ethical standards, ensuring the confidentiality, privacy, and security of patient information.

Patients who underwent telemedicine follow-up had previously signed an informed statement acknowledging and agreeing to this new model of care.

### Eligibility Criteria

Inclusion criteria were presence of a tracheal device; aged ≥18 years; regular follow-up at the Trachea and Airway Outpatient Clinic of the Division of Thoracic Surgery, Hospital das Clinicas, University of Sao Paulo; signed informed statement acknowledging and agreeing to online consultation; and to participate in a telemedicine session with simultaneous audio and video.

### Study Setting and Telemedicine Platform

**Figure 1.**
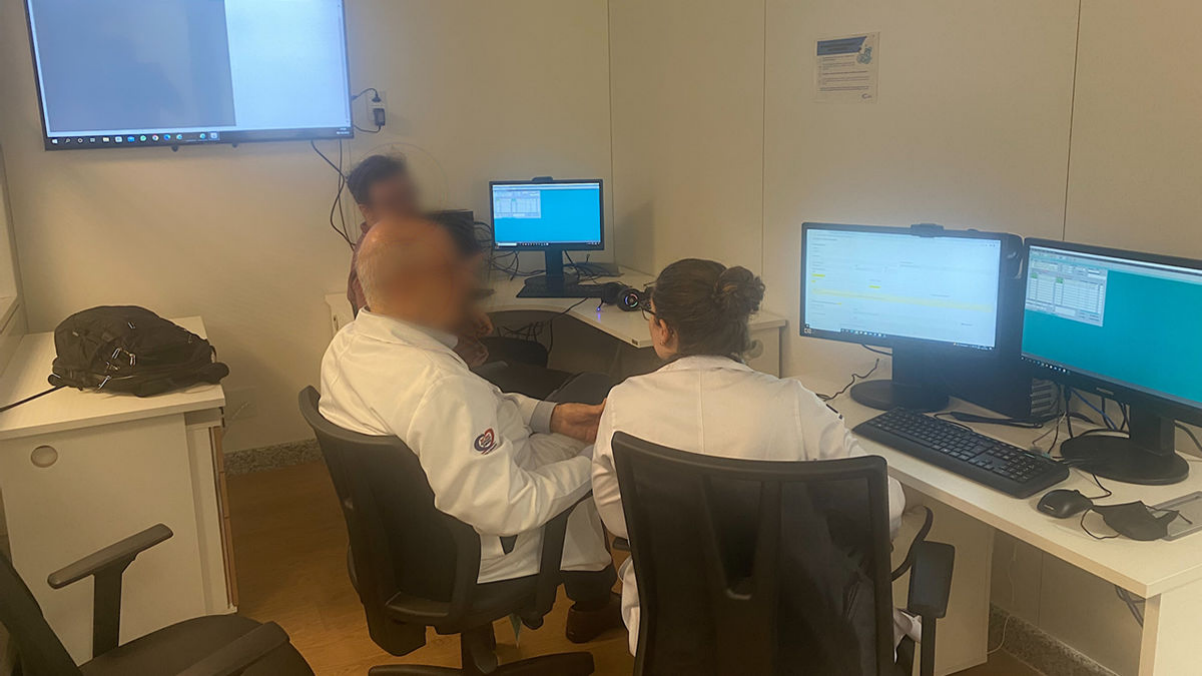
Telemedicine consultation room with desktop computers with cameras, headsets, and integrated microphones. Teleconsultations were conducted by residents and fellows under the supervision of a senior staff thoracic surgeon.

**Figure 2.**
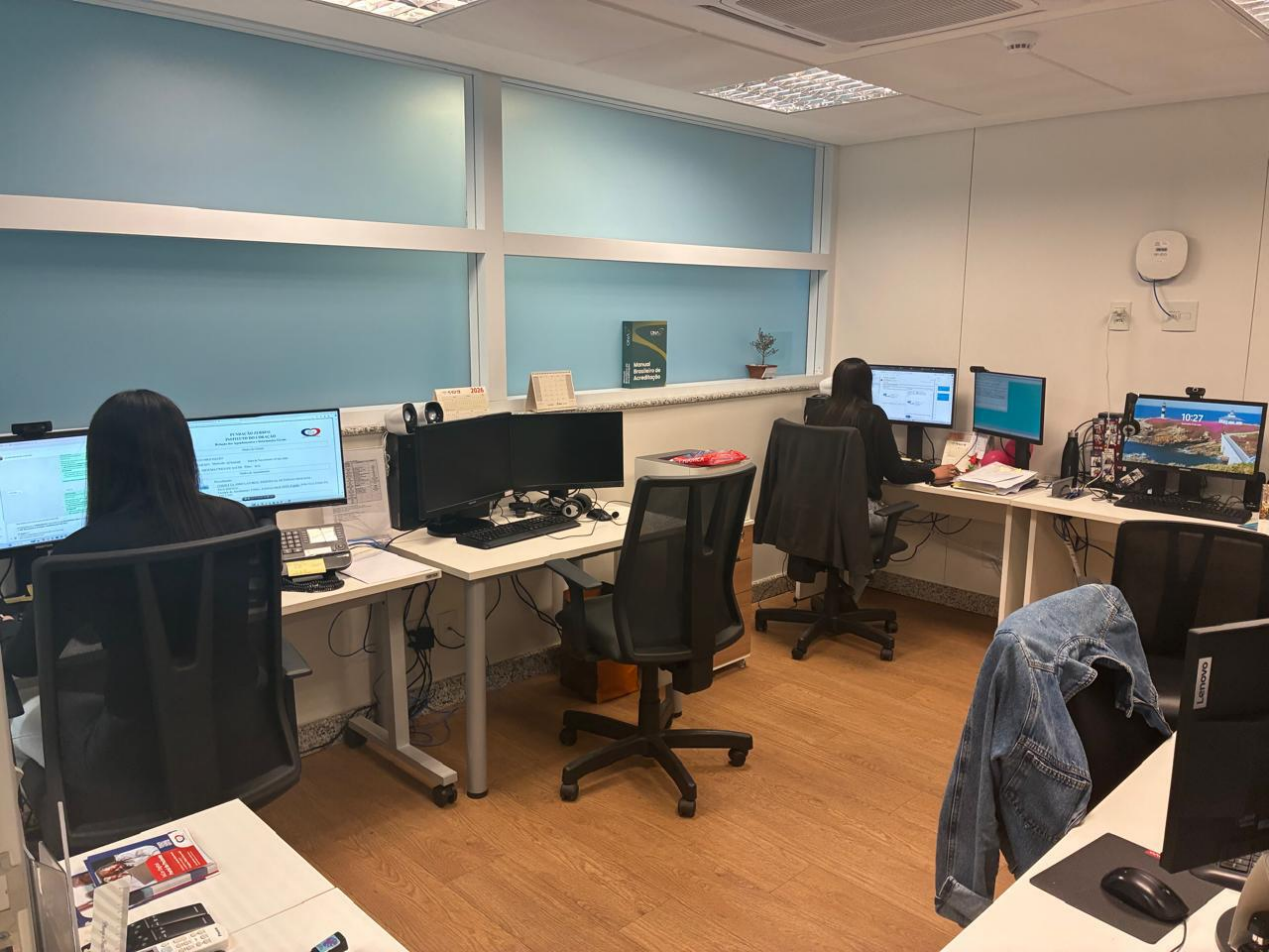
The telemedicine support team workspace is adjacent to the medical team’s teleconsultation room.

Telemedicine consultations were conducted at the InCor-HCFMUSP. The facility has 14 soundproof teleconference rooms, each equipped with desktop computers with dual monitors and headsets with integrated microphones (Figure 1). The support team, located in an adjacent room, provided technical assistance in the event of connection disruptions or audiovisual difficulties (Figure 2) [17,18].

The online consultations were conducted by thoracic surgery residents and fellows under the on-site supervision of a senior staff thoracic surgeon (PFGC and PPDC). The supervising surgeon reviewed and discussed each case and was available to interact directly with the patient and caregiver when necessary.

**Figure 3.**
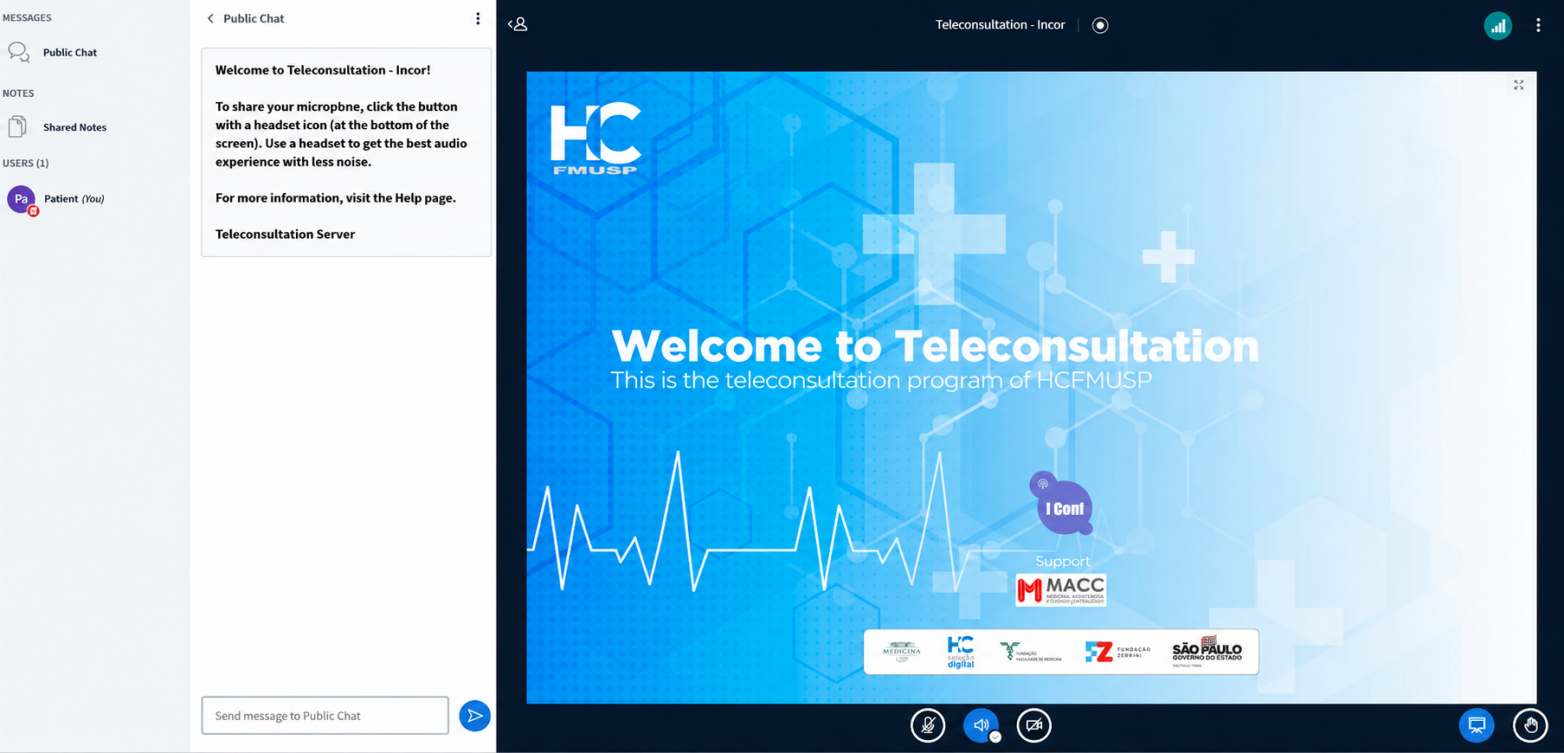
The telemedicine institutional platform interface is used for teleconsultations.

The teleconsultation process used the hospital’s proprietary institutional teleconferencing platform (iConf, InCor-HCFMUSP; Figure 3). This system was designed to guarantee the secure storage and confidentiality of all service-related data within the institution with no access or manipulation by third parties [21,22]. Digital consultations were documented in the hospital’s proprietary electronic medical record system (Si3, InCor-HCFMUSP). This integrated platform consolidates data from outpatient visits, hospital admissions, laboratory, and imaging results. It provides comprehensive access to recent and historical information supporting continuity and coordination of patient care.

### Definitions of Study Outcomes

Outcomes were identified through a review by the principal investigator to confirm eligibility for each outcome category. The electronic medical records, according to the predefined study definitions, ED visits, hospital readmissions, 30-day hospital readmissions, and unplanned interventions were classified according to their association with tracheal disease or device complications.

The primary outcome was whether patients visited the ED during the follow-up period. This was defined as any ED visit motivated by an airway-related symptom (dyspnea, stridor, or infection such as aspiration pneumonia or tracheitis), or for tracheal device–related issues (device fracture, accidental decannulation, loss of tracheostomy cuff pressure, or the need to keep the lateral limb of the T-tube open).

The secondary outcomes were (1) readmissions (defined as unplanned admissions for any clinical deterioration related to the tracheal device occurring during the study period; complications unrelated to the tracheal device were not considered); (2) 30-day readmissions (defined as unplanned admissions for any any clinical deterioration related to the tracheal device occurring within 30 days of the last outpatient visit; complications unrelated to the tracheal device were not considered); and (3) unplanned interventions (defined as any nonscheduled airway procedure during the study period, such as the need to replace a damaged tracheal device, device migration, obstruction, granulation tissue, or bleeding requiring bronchoscopy).

### Statistical Analysis

Patient characteristics were summarized as absolute and relative frequencies (%) for categorical variables, and as mean with SD, and median with minimum and maximum values, or IQR for continuous variables. Categorical variables were examined by Pearson chi-square test or Fisher exact test, and continuous variables by the Mann-Whitney *U* test. For paired comparisons, McNemar test was used for categorical variables and the Wilcoxon signed-rank test for continuous variables.

Propensity score matching (PSM) balanced baseline characteristics using a binary logistic regression model with age, race, type of tracheal device, and diagnosis as covariates. The matched cohort was generated via 1:1 nearest-neighbor matching using a caliper of 0.05, and covariate balance before and after matching was assessed using standardized mean differences (Multimedia Appendix 1). Odds ratios with 95% CIs were estimated using logistic regression to compare the occurrence of ED visits, hospital readmissions, 30-day hospital readmissions, and unplanned interventions between groups after PSM.

For the PSM analyses, outcomes were treated as binary variables, defined by the occurrence of at least 1 event per patient during follow-up. The number of readmissions and unplanned interventions per patient was additionally summarized descriptively. For time-to-event analysis, Kaplan-Meier curves were used to estimate ED visit-free; only the first ED visit was considered and defined as the interval from the outpatient visit to the ED visit. Curves were compared between groups using the log-rank test after PSM.

All statistical tests were 2-sided, with a significance level set at 5%. Analyses were performed using SPSS for Windows (version 25; IBM Corp).

## Results

**Figure 4.**
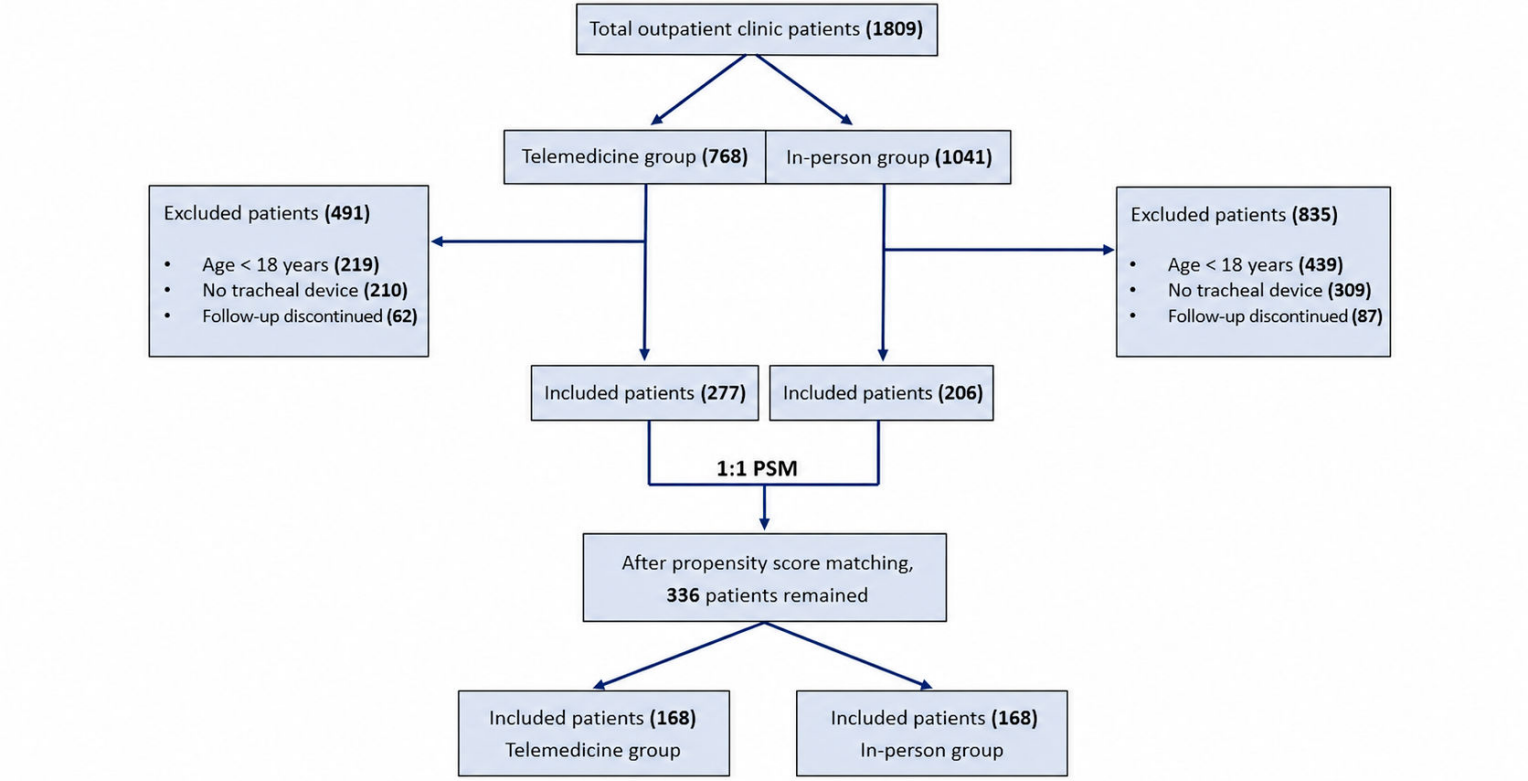
Flowchart of patient selection and propensity score matching. PSM: propensity score matching.

The number of patients is depicted in Figure 4. During the study, 483 patients were included (n=277, 57% telemedicine vs n=206, 43% in-person), with a total of 2487 visits (1258 telemedicine vs 1229 in-person). All patients had a tracheal device (246 tracheostomies, 192 silicone T-tubes, and 45 endoprostheses). The commonest diagnoses were postintubation tracheal stenosis (n=332, 69%), followed by neurological diseases (n=100, 21%), idiopathic stenosis (n=13, 3%), and tracheobronchial tuberculosis (n=12, 2.5%). The mean patient age was 46 (SD 16 years; range 18‐92 years), and the Charlson comorbidity index score mean was 2 (SD 2).

Baseline characteristics are summarized in Table 1. Before matching, the telemedicine group had a higher proportion of patients with silicone T-tubes, whereas tracheostomy was more frequent in the in-person group. Significant differences between the groups were also observed for race and diagnosis, while the remaining baseline variables were generally comparable.

**Table 1.** Baseline demographics and clinical characteristics of adult patients with tracheal devices before propensity score matching.

	Group		*P* value
	In person (n=206)	Telemedicine (n=277)	
Sex, n (%)			.92
Female	95 (46.1)	129 (46.6)	
Male	111 (53.9)	148 (53.4)	
Age (years), mean (SD)	48.1 (17.1)	45.5 (16.5)	.09
Race, n (%)			<.001
White	158 (76.7)	249 (89.9)	
Brown	25 (12.1)	18 (6.5)	
Black	23 (11.2)	10 (3.6)	
Education, n (%)			.15
College graduate	21 (11.6)	46 (17.6)	
High school graduate	63 (34.8)	88 (33.7)	
Less than high school	89 (49.2)	120 (46.0)	
No schooling	8 (4.4)	7 (2.7)	
Tracheal device, n (%)			<.001
Tracheostomy	132 (64.1)	114 (41.2)	
Silicone T-tube	58 (28.2)	134 (48.4)	
Endoprosthesis	16 (7.8)	29 (10.5)	
Diagnosis, n (%)			.04
Orotracheal intubation	129 (62.6)	203 (73.3)	
Neurologic diseases	57 (27.7)	43 (15.5)	
Idiopathic diseases	7 (3.4)	6 (2.2)	
Tuberculosis	4 (1.9)	8 (2.9)	
Neoplasm	4 (1.9)	6 (2.2)	
Rheumatologic diseases	3 (1.5)	5 (1.8)	
Others	2 (1.0)	6 (2.2)	
Charlson index, mean (SD)	2.0 (1.9)	2.0 (2.1)	.86
Charlson index (severity), n (%)			.81
Low risk	136 (66.0)	190 (68.6)	
Intermediate risk	44 (21.4)	53 (19.1)	
High risk	26 (12.6)	34 (12.3)	

A total of 140 patients (29%) required ED visits. Patients with tracheal endoprostheses had a higher rate of ED visits than patients with silicone T-tubes or tracheostomies. Patients with tracheobronchial tuberculosis sought ED more frequently when compared to other etiologies. There was no significant difference between the telemedicine and in-person groups (n=85, 30.7% vs n=55, 26.7%; *P*=.34; Table 2).

**Table 2.** Baseline demographics and clinical characteristics according to emergency department visits among adult patients with tracheal devices before propensity score matching.

	Emergency department visits		*P* value
	No (n=343)	Yes (n=140)	
Sex, n (%)			.10
Female	151 (67.4)	73 (32.6)	
Male	192 (74.1)	67 (25.9)	
Age (years), mean (SD)	48.1 (17.1)	45.5 (16.5)	.42
Race, n (%)			.11
White	286 (70.3)	121 (29.7)	
Brown	36 (83.7)	7 (16.3)	
Black	21 (63.6)	12 (36.4)	
Education, n (%)			.30
College graduate	43 (64.2)	24 (35.8)	
High school graduate	99 (65.6)	52 (34.4)	
Less than high school	151(72.2)	58(27.8)	
No schooling	13 (86.7)	2 (13.3)	
Tracheal device, n (%)			<.001
Tracheostomy	198 (80.5)	48 (19.5)	
Silicone T-tube	119 (62.0)	73 (38.0)	
Endoprosthesis	26 (57.8)	19 (42.2)	
Diagnosis, n (%)			.01
Orotracheal intubation	222 (66.9)	110 (33.1)	
Neurologic diseases	85 (85.0)	15 (15.0)	
Idiopathic diseases	10 (76.9)	3 (23.1)	
Tuberculosis	7 (58.3)	5 (41.7)	
Neoplasm	7 (70.0)	3 (30.0)	
Rheumatologic diseases	5 (62.5)	3 (37.5)	
Others	7 (87.5)	1 (12.5)	
Charlson index, mean (SD)	2.0 (1.9)	2.0 (2.1)	.95
Charlson index (severity), n (%)			.36
Low risk	234 (71.8)	92 (28.2)	
Intermediate risk	71 (73.2)	26 (26.8)	
High risk	38 (63.3)	22 (36.7)	
Group, n (%)			.34
In-person	151 (73.3)	55 (26.7)	
Telemedicine	192 (69.3)	85 (30.7)	

Regarding unplanned readmissions and interventions, there were no statistically significant differences between the groups. Conversely, the 30-day hospital readmissions were significantly lower in the telemedicine group (n=18, 21.2% vs n=27, 49.1%, *P*=.001; mean 0.2, SD 0.4 vs mean 0.7, SD 0.8, *P≤*.001; Table 3).

**Table 3.** Hospital readmission, 30-day hospital readmission, and unplanned interventions outcomes before propensity score matching in adult patients with tracheal devices.

	Group		*P* value
	In person (n=55)	Telemedicine (n=85)	
Readmission, n (%)			.25
No	23 (41.8)	44 (51.8)	
Yes	32 (58.2)	41 (48.2)	
Readmissions, mean (SD)	0.8 (0.8)	0.5 (0.6)	.10
30-day hospital readmission, n (%)			.001
No	28 (50.9)	67 (78.8)	
Yes	27 (49.1)	18 (21.2)	
30-day hospital readmissions, mean (SD)	0.7 (0.8)	0.2 (0.4)	<.001
Unplanned intervention, n (%)			.83
No	21 (38.2)	31 (36.5)	
Yes	34 (61.8)	54 (63.5)	
Unplanned interventions, mean (SD)	0.9 (0.9)	0.8 (0.9)	.66

**Figure 5.**
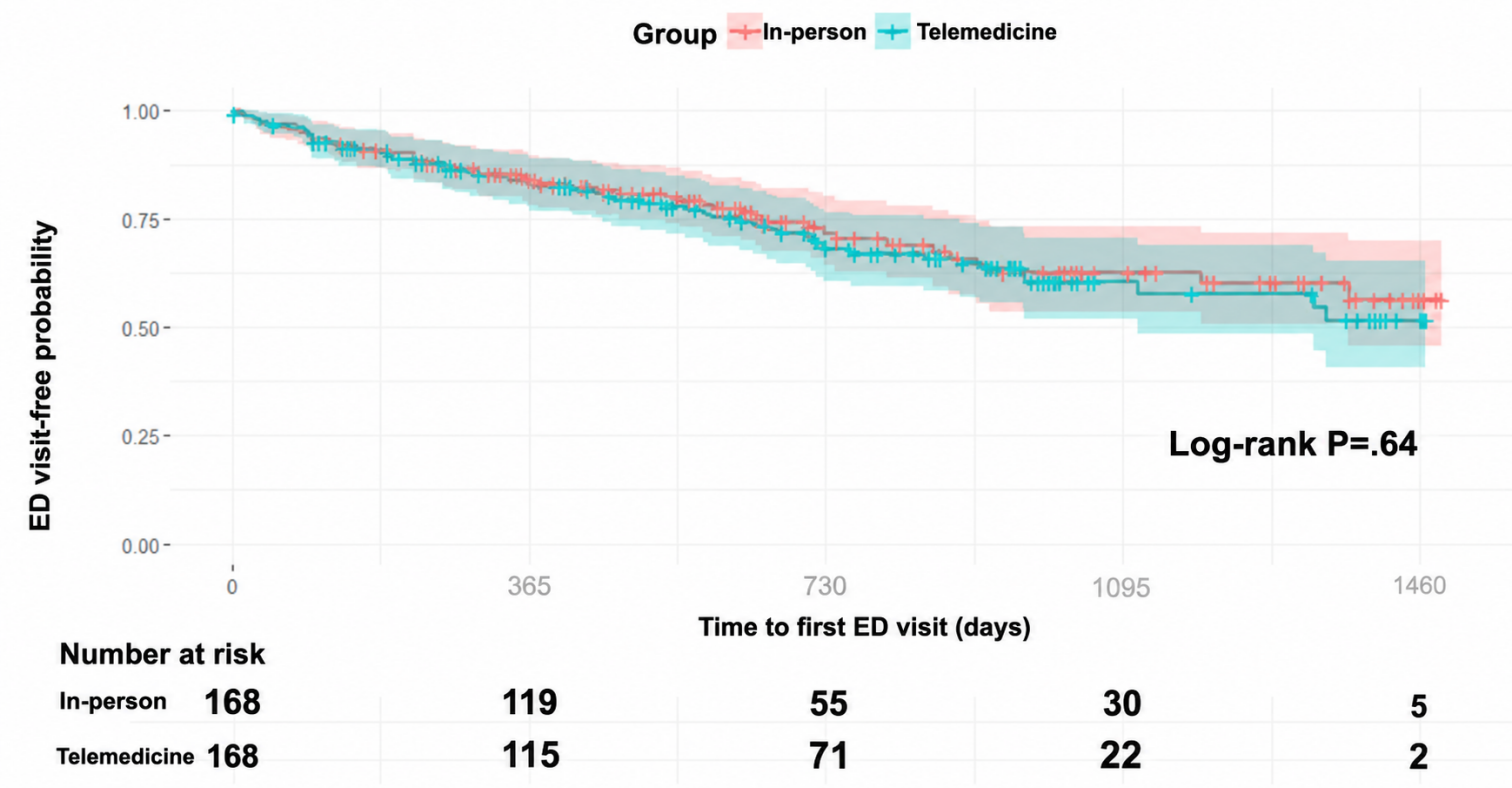
Kaplan-Meier curve for time to first emergency department (ED) visit after propensity score matching.

After PSM, there were 168 patients per group (Table 4). The logistic regression analysis showed no significant differences between the groups in ED visits, hospital readmissions, or unplanned interventions. However, patients in the telemedicine group had significantly fewer 30-day hospital readmissions (odds ratio 0.38, 95% CI 0.16‐0.87; *P*=.02; Table 5). The Kaplan-Meier analysis showed no statistically significant difference between the groups (*P*=.64), with no meaningful separation between the curves over time (Figure 5).

**Table 4.** Baseline demographics and clinical characteristics of adult patients with tracheal devices after propensity score matching.

	Group		*P* value
	In person (n=168)	Telemedicine (n=168)	
Age (years), mean (SD)	48.2 (17.2)	47.5 (17.0)	.67
Race, n (%)			.27
White	152 (90.5)	141 (83.9)	
Brown	9 (5.4)	17 (10.1)	
Black	7 (4.2)	10 (6.0)	
Tracheal device, n (%)			.58
Tracheostomy	103 (61.3)	90 (53.6)	
Silicone T-tube	49 (29.2)	60 (35.7)	
Endoprosthesis	16 (9.5)	18 (10.7)	
Diagnosis, n (%)			.62
Orotracheal intubation	105 (62.5)	113 (67.3)	
Neurologic diseases	49 (29.2)	35 (20.8)	
Tuberculosis	4 (2.4)	5 (3.0)	
Idiopathic diseases	5 (3.0)	3 (1.8)	
Neoplasm	2 (1.2)	5 (3.0)	
Rheumatologic diseases	2 (1.2)	3 (1.8)	
Others	1 (0.6)	4 (2.4)	

**Table 5.** Logistic regression analysis of clinical outcomes after propensity score matching in adult patients with tracheal devices followed by telemedicine or in-person care.

Variables	OR^a^ (95% CI)	*P* value
Emergency department visit	1.19 (0.74‐1.90)	.47
Readmission	0.70 (0.31‐1.54)	.37
30-day hospital readmission	0.38 (0.16‐0.87)	.02
Unplanned intervention	1.73 (0.74‐4.04)	.20

a OR: odds ratio.

## Discussion

### Principal Findings

This single-center, propensity score–matched cohort of adult patients with tracheal devices demonstrated that telemedicine follow-up yielded safety outcomes comparable to those of in-person care. Following PSM, there were no significant differences between groups for ED visits, hospital readmissions, or unplanned interventions. Thirty-day hospital readmissions were significantly lower in the telemedicine group. The present findings suggest that a structured telemedicine program may support the longitudinal management of patients with tracheal devices without increasing ED visits. In the matched analysis, telemedicine follow-up was also associated with lower odds of 30-day hospital readmission.

Building on these findings, we must situate our results within the well-documented burden of tracheal device–related complications on patients and health systems [23]. Complications associated with tracheal devices result in substantial emergency care costs. In a recent analysis, tracheostomy-related complications accounted for 6156 ED visits and 2316 readmissions in a single year, with median inpatient charges of US $66,416 [24]. Our study showed no significant differences between the telemedicine and in-person groups in ED visits, but lower 30-day readmissions in the telemedicine group. These findings suggest that adequate remote surveillance may help prevent clinical deterioration and reduce downstream costs.

Studies on follow-up via telemedicine demonstrate that the quality of care is not inferior, regardless of the ED visits and readmissions [25-28]. Telemedicine in thoracic surgery cohorts has been associated with favorable outcomes [29-31]. The study by Xiao et al [31] reported that after esophagectomy, telemedicine follow-up resulted in fewer ED visits than in-person follow-up (mean 0.45, SD 0.76 vs 0.79, SD 1.12 within 6 months; *P*=.03), supporting the potential of telemedicine.

During the telemedicine visit, patients wearing tracheal devices require a visual inspection, as well as an audio capability that may prevent real-time communication. This context can undermine the quality of telemedicine by increasing the risk of misdiagnosis, requiring an ED visit. In this regard, our program offered synchronous audio-video consultations to enable patient and caregiver engagement, standardized checklists for device assessment, and provided technical support for transmission issues. So far, we have not observed worse outcomes, as shown by the absence of significant differences in ED visits, unplanned readmissions, and interventions.

Hospital readmissions affect patients’ survival and quality of life, strain the health care resources, and serve as a benchmark of hospital performance [32-35]. Across health care payers, the all-cause 30-day readmission rate is 14% [36]. Although some readmissions are unavoidable, many are unplanned, and approximately 30% are preventable [37]. Therefore, reducing readmissions has become a key objective [38] to lower costs and improve the quality of care [39]. Recent studies have demonstrated a reduction in the risk of 30-day readmissions for patients who underwent telemedicine follow-up [40-42].

Unlike prior studies focused primarily on the immediate postdischarge period, our analysis focused on the outpatient follow-up setting. We hypothesized that telemedicine for outpatient follow-up in patients with tracheal devices may exert distinct and durable effects on safety and device use.

An important point for readmission in our patients is that visits exert a significant impact immediately after the consultation, when preventable failures such as early diagnosis of infection, medication or equipment mismanagement, and caregiver uncertainty are most likely to occur. However, beyond 30 days, the readmissions are mainly driven by the tracheal disease trajectory and factors related to device deterioration. Our study showed a reduction in 30-day readmissions with telemedicine follow-up, and no significant differences were found after this period. However, this finding should be interpreted with caution. The retrospective design does not allow causal inference. Unmeasured confounders such as differences in patient engagement, caregiver availability during follow-up, or clinician triage behavior may have partially contributed to the lower 30-day readmission rate observed in the telemedicine group, although this finding remains clinically relevant.

Other features of in-person follow-up may also influence the outpatient experience, including travel to the medical center, long waiting times [43,44], and the dynamics of a busy academic environment. In contrast, telemedicine consultations in our program were delivered through synchronous audio-video encounters within a structured workflow that included previsit technical support and postvisit review by the telemedicine team, as previously reported [17,18]. These factors may have contributed to continuity of care and reinforcement of recommendations during the follow-up period. In addition, prior studies have suggested that receiving care in the home environment may improve patient comfort and reduce stress during telemedicine visits [45,46]. Although these factors were not directly measured in our study, they may also represent plausible contributors to the lower 30-day readmission rate observed in the telemedicine group.

This study has several limitations. It is performed at a single high-volume center for tracheal diseases with a dedicated airway surgery team, which can limit external generalizability. Despite the planned data collection, the study analyses are retrospective, and even after PSM, the groups can still exhibit selection bias.

Although telemedicine was systematically offered, follow-up modality was not assigned at random. Patients who remained in the in-person group often did so because of limited digital access, lack of caregiver support, or personal preference, factors that may reflect broader differences in social support, health engagement, and adherence to follow-up. These variables may have influenced outcomes even after PSM.

During the COVID-19 pandemic, our academic hospital actively promoted telemedicine by investing in a high-complexity infrastructure and comprehensive staff training to ensure successful implementation and its continuity after the pandemic. Our findings reflect a mature, well-resourced perennial program, which may also limit generalizability to settings without comparable resources or experience. Nevertheless, certain elements of the model are likely essential regardless of setting, including a structured consultation environment with adequate computers and reliable audio-video equipment, technical team support for both patients and clinicians, and continued training in digital care.

Beyond the direct comparison of outcomes, our findings suggest a broader role for structured telemedicine in the longitudinal management of patients with tracheal devices. As these patients often require ongoing specialized follow-up and are frequently concentrated in referral centers, telemedicine may help reduce geographic and logistical barriers to care while maintaining continuity of monitoring. Although these results should be interpreted within the limits of a retrospective single-center study, they support the integration of structured telemedicine pathways into the care of selected patients with tracheal devices and provide a rationale for future prospective evaluation in other settings.

### Conclusions

In this cohort of adult patients with tracheal devices, telemedicine outpatient follow-up was associated with clinical outcomes comparable to those of in-person follow-up. Telemedicine follow-up showed similar ED visit rates and was associated with lower odds of 30-day hospital readmission in the matched analysis.

## Supplementary material

10.2196/95479 Multimedia Appendix 1 Absolute standardized mean differences before and after propensity score matching.

## Abbreviations

ED: emergency department
InCor-HCFMUSP: Heart Institute of Hospital das Clinicas, University of Sao Paulo
PSM: propensity score matching
