# Supplementary material for: Safety of Telemedicine Versus In-Person Care for Patients With Tracheal Devices: Propensity Score–Matched Cohort Study
Source: J Med Internet Res. 2026 May 26;28:e95479. doi: 10.2196/95479 (PMC13204319; doi:10.2196/95479)
Supplement: Multimedia Appendix 1 [file jmir-v28-e95479-s001.docx]

**Supplementary File 1. Absolute standardized mean differences before and after propensity score matching**

| **Variable** | **Before PSM** | **After PSM** |
| --- | --- | --- |
| **Age** | 0.154 | 0.100 |
|  |  |  |
| **Race** |  |  |
| White | 0.359 | 0.017 |
| Brown | 0.195 | 0.021 |
| Black | 0.292 | 0.000 |
|  |  |  |
| **Tracheal device** |  |  |
| Tracheostomy | 0.472 | 0.135 |
| Silicone T-tube | 0.425 | 0.199 |
| Endoprosthesis | 0.094 | 0.077 |
|  |  |  |
| **Diagnosis** |  |  |
| Orotracheal intubation | 0.230 | 0.051 |
| Neurologic diseases | 0.298 | 0.056 |
| Tuberculosis | 0.062 | 0.090 |
| Idiopathic diseases | 0.028 | 0.000 |
| Neoplasm | 0.016 | 0.037 |
| Rheumatologic diseases | 0.096 | 0.049 |
| Others | 0.075 | 0.000 |
